# Temporal Evolution of the Profile of Patients Hospitalized with Heart Failure (2000–2022)

**DOI:** 10.3390/clinpract15100187

**Published:** 2025-10-16

**Authors:** Teresa Seoane-Pillado, Roi Suárez-Gil, Sonia Pértega-Díaz, Juan Carlos Piñeiro-Fernández, Elena Rodriguez-Ameijeiras, Emilio Casariego-Vales

**Affiliations:** 1 Rheumatology and Health Research Group, Department of Health Sciences, Faculty of Health Sciences, University of La Coruña, Campus de Oza, 15006 La Coruña, Spainsonia.pertega.diaz@sergas.es (S.P.-D.); 2 Nursing and Health Care Research Group, Biomedical Research Institute of La Coruña (INIBIC), Xubias de Arriba 84, 15006 La Coruña, Spain; 3 Internal Medicine Department, Lucus Augusti University Hospital, C/Dr. Ulises Romero, s/n, 27003 Lugo, Spainjuan.carlos.pineiro.fernandez@sergas.es (J.C.P.-F.);; 4 Internal Medicine Department, University Hospital Complex of Santiago, C/Choupana s/n, 15076 Santiago de Compostela, Spain

**Keywords:** heart failure, temporal trends, multimorbidity

## Abstract

**Background:** The clinical characteristics of patients who have a first episode of congestive heart failure (CHF) may have changed in recent years. **Methods:** A retrospective cohort study was performed on 19,796 patients discharged from medical departments with a diagnosis of CHF between 1 January 2000 and 31 December 2022. Data were drawn from two data sets of the Minimum Basic Data Set-Hospital Data Set (MBDS) of the Lucus Augusti University Hospital (Spain): hospitalizations and patients. Patient characteristics (including the period of their first admission) and the association rules between diseases determined using the Apriori algorithm were studied in five consecutive time periods. **Results:** The general characteristics of patients on first admission for CHF changed over time. There were increases in mean age (75.9 ± SD 11.2 vs. 81.6 ± SD 11.5 years; *p* < 0.0001), the proportion of women (48.3% vs. 51.4; *p* = 0.0001), the number of acute diseases (1.1 ± SD 1.4 to 2.7 ± SD 2.5; *p* < 0.0001), and the number of chronic diseases (3.6 ± SD 1.9 to 6.5 ± SD 2.6); *p* < 0.001). Accordingly, the median number of diagnoses (from 3 to 7) and itemsets per patient increased (mean number of items 1.75 vs. 3.4; *p* < 0.0001), and the associations of diseases leading to CHF became more complex. **Conclusions:** This single-center study shows that in the last two decades, the characteristics of patients with a first hospital admission for CHF have changed. Patients are older, there is a predominance of women, and they have a greater number of acute and chronic concomitant diseases, making their clinical management more difficult.

## 1. Introduction

Congestive heart failure (CHF) is a highly complex clinical syndrome. Its signs and symptoms are the consequence of structural or functional disorders of ventricular filling or ejection. Although multiple diseases (e.g., ischemic heart disease, diabetes, etc.) or habits (sedentary lifestyle, etc.) are associated with CHF, the risk they pose for developing this condition varies greatly [1]. Moreover, the associated diseases usually do not occur individually, but rather multimorbidity is more common. When the different conditions are not only associated but also interact with each other, the complexity of the clinical approach and treatment is even greater [2]. Notable changes have been observed in the multimorbidity of Spanish patients, due in part to population aging in recent decades [3,4,5,6]. This undoubtedly entails changes in the presentation of CHF and other diseases. However, little is known about variations in the clinical presentation of CHF, changes in these patients’ disease burden, and the influence these factors may have on readmission rates or mortality [7,8,9]. Identifying variations in patients, their underlying diseases, and associations among diseases is useful for understanding the pathophysiological mechanisms of CHF onset and improving preventive or therapeutic strategies.

Data mining has been used in different disciplines to extract information from large amounts of data. In medicine, it has been used in areas as diverse as searching for risk factors or determining patients’ needs. Given its results, it is considered a suitable technique for classifying patients and generating predictions [10,11]. This study used data mining techniques to evaluate which diseases or clusters of diseases are associated with CHF and analyze how they have evolved over this century.

## 2. Materials and Methods

This work is a retrospective cohort study on all patients diagnosed with CHF during hospitalization in any adult medical department of the Lucus Augusti University Hospital of Lugo, Spain between 1 January 2000 and 31 December 2022.

During the study period, the center had 769 beds distributed among three hospital centers and provided care for a population of 240,000 inhabitants. This hospital complex was closed in December 2010. All resources and equipment from the three hospital centers were transferred to a new building, the Lucus Augusti University Hospital, which has 879 beds. During both periods, the medical area comprised the following 12 departments: Cardiology, Endocrinology, Rheumatology, Oncology, Respiratory Medicine, Gastroenterology, Neurology, Nephrology, Geriatric Medicine, Infectious Diseases, and Internal Medicine.

The data source was the center’s minimum basic data set (MBDS), a mandatory registry of clinical information recorded in patients’ electronic medical records. Patients with a diagnosis of CHF were selected. The variables analyzed included the hospitalization department, sex (male/female), date of birth, hospital admission and discharge date, length of stay (days), destination at discharge, diagnosis-related group (DRG) for the principal diagnosis, and secondary diagnoses in order of appearance. It is mandatory for all hospitals in Spain to input these variables for each admission and discharge. Therefore, data were available in all cases, and no patients were excluded due to missing data. The original data were entered by the attending healthcare professional and coded by experienced professionals. The International Classification of Diseases, Ninth Revision, Clinical Modification (ICD-9-CM) was used until 2015, when it was replaced by the ICD-10-CM. Nursing registries were used as additional data sources and, since 2007, the IANUS computerized database was also used, which gathers all data from medical care. The database was reviewed for errors such as incomplete data, classification errors, typing errors, etc. Then, all cases were verified individually, checking the medical record when necessary, until the database was considered error-free. An electronic database of each hospitalization event using all the aforementioned registries was created. It includes all hospitalizations and their associated principal and secondary diagnoses thought to be the cause of hospitalizations. Given the absence of a widely agreed-on list of chronic diseases, a modified version of the German MultiCare study adapted to the inpatient context was used [3]. Under this method, 32 common chronic diseases were defined. Finally, a second classification of acute diseases was created. To determine which should be included, a qualified physician defined them as diseases that had been ongoing for less than 30 days. Once this first database was completed, a second database was generated in which patient data were analyzed. It included the main study variables (presence/absence of the 32 chronic diseases) as well as secondary variables such as gender, date of birth, and hospitalization dates.

The study protocols were approved by the Clinical Research Ethics Committee of Galicia (CREC of Galicia registry code 2022/372).

### Data Analysis

A descriptive analysis of the variable was performed according to the time period. R software was used to perform an association rule analysis using the Apriori algorithm to identify patterns in the comorbidities of patients with CHF using the R package ‘arules V 0.4-2’ [12,13]. The dataset was classified into five time periods (2000–2004; 2005–2009; 2010–2014; 2015–2019; and 2020–2022). Follow-up was conducted by analyzing the administrative records with the censoring date of 31 March 2023. Each period included information on patients hospitalized for the first time in that period with follow-up until their last admission. Each record contained sociodemographic and clinical information. The medical record number was used as a unique identifier so as to connect multiple hospitalizations of the same patient and build a database. The data were preprocessed before applying the Apriori algorithm.

Each sequence of diagnosis codes at the time of medical discharge was referred to as a transaction. Within a transaction, each diagnosis recorded was an item, and each possible combination of items in the transaction was called an itemset. The Apriori algorithm aims to extract the association rules for each set of items within each transaction. A rule “itemset1 (antecedent) → itemset2 (consequent)” indicates that the presence of itemset1 in a transaction implies the presence of itemset2 in the same transaction. However, it merits mention that the rules derived by the Apriori algorithm do not necessarily establish a cause-and-effect relationship between itemsets.

The key parameters in association rules are support and confidence. Support indicates the relative frequency of comorbidities in the data set. Confidence is the conditional probability of the occurrence of itemset2 given itemset1. That is, the percentage of transactions containing both itemset1 and itemset2 in all transactions containing itemset1. A third parameter, lift, measures the independence between itemset1 and itemset2. A value greater than 1 indicates that the antecedent itemset and the consequent itemset are dependent and positively correlated. A value less than 1 indicates that the two itemsets are independent and there is a weak association between them.

In this work’s analysis, various minimum thresholds for the support and confidence parameters were tested so as to identify the most significant association rules in each period. Selecting these thresholds presented certain challenges, as they varied according to the dataset’s specific context. Setting extremely high thresholds often resulted in the omission of relevant information. Finally, minimum values of 1% for support, 50% for confidence, and a lift greater than 1 were established. Among the rules generated, those meeting these criteria whose antecedents contained a maximum of four elements (ICD-9-CM codes) with a single consequent, also coded according to ICD-9-CM, were selected and ordered according to confidence level. This selection was made to avoid an excessively dispersed representation that could result from having a large number of elements in both antecedents and consequents, which could lead to less meaningful representation of the population.

## 3. Results

**Figure 1:**
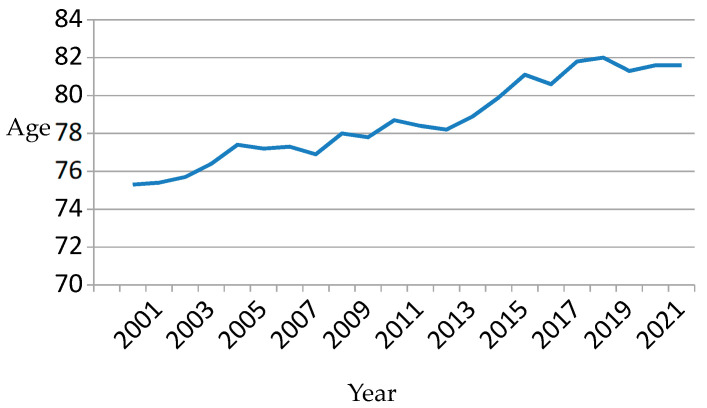
Evolution of the average age of patients on their first hospital admission.

**Figure 2:**
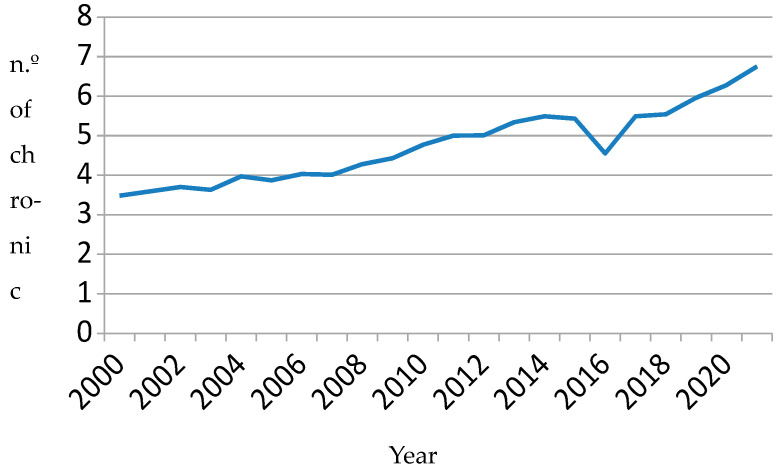
Mean number of chronic conditions detected during the patient’s first admission.

Between 2000 and 2022, 19,727 patients were admitted at least once for CHF. Table 1 shows patients’ clinical characteristics in the five consecutive time periods. Each patient was assigned to the period of the first CHF admission. Patients’ general characteristics changed over time. For example, the mean age increased from 75.9 (SD = 11.2) in the 2000–2004 period to 81.6 (SD = 11.5) in the 2020–2022 period (*p* = 0.0001). The percentage of men decreased significantly (51.7% in the first period versus 48.6% in the last; *p* = 0.0001). There were also significant increases in the mean number of acute diseases (1.1 (SD = 1.4) to 2.7 (SD = 2.5)) and chronic diseases (3.6 (SD = 1.9) to 6.5 (SD = 2.6)) (*p* < 0.001 in both cases). In summary, over time, patients on their first admission for CHF were increasingly older, a higher proportion were women, and they had a greater burden of multimorbidity of both acute and chronic diseases. Changes in average age and chronic conditions can be seen in Figure 1 and Figure 2, respectively.

Table 2 shows that over the 22 years of the study, the number of total diagnoses remained relatively stable (252 in the first period to 207 in the last period, which included two fewer years). However, the median number of diagnoses per patient (3 to 7) and number of itemsets increased. The itemsets also became progressively more complex, including a greater number of items (1.75 to 3.4; *p* < 0.0001).

An analysis of association rules using the Apriori algorithm allowed for identifying the most probable patterns that developed as a consequence of heart failure (HF) in the different periods (Table 3) (see Appendix Table A1 for the complete tables). Between 2000 and 2004, the association between cardiomyopathy and CHF was notable (confidence 72.08% and lift 1.59). Relevant associations were also identified: pulmonary hypertension, valvular heart disease, arrhythmias, chronic obstructive pulmonary disease, type 2 diabetes mellitus, and acute renal failure were frequent antecedents of CHF.

In 2005–2009, combinations such as renal and respiratory failure (confidence 75.8%, lift 1.55) or valvular heart disease and pulmonary hypertension stood out, with a confidence of 74.6%. There was a greater presence of arrhythmias, cardiomyopathies, and respiratory disorders in CHF cases, reflecting a more complex clinical profile. This trend toward greater diagnostic complexity continued between 2010 and 2014, with a notable link between atrial fibrillation and metabolic disorders. Associations were also observed between hypertension, valvular heart disease, diabetes, obesity, arrhythmias, and progression to CHF.

In 2015–2019, multiple high-confidence rules were identified that consolidated the aforementioned relationships. In 2020–2022, complex combinations persisted, such as arrhythmias and heterogeneous problems (confidence 100%, support 5.26%), ischemic heart disease and renal failure (support 3.77%), and hypertension and arrhythmias (support 3.12%). This showed a trend toward a more complex clinical profile and an increasing burden of comorbidity.

## 4. Discussion

This study shows that from 2000 to 2022, the characteristics of patients with CHF hospitalized for the first time have changed substantially. First, there has been a gradual increase in age and a consequent—and growing—complexity of the first hospital admission. Second, there is an increasing predominance of women. Third, the diseases leading to CHF have changed in part over time. Finally, although the number of underlying diseases is small, they tend to combine in increasingly numerous and heterogeneous groups and their management has become progressively more difficult. In summary, patients with CHF have changed and their clinical management is increasingly complex.

It is well known that CHF results from the variable sum of different diseases that decompensate the patient, either by gradual worsening or by an external factor that triggers decompensation. Therefore, cardiovascular and non-cardiovascular multimorbidity and frailty are becoming more common, resulting in highly varied and complex clinical pictures [8]. Logically, a patient’s age is a very relevant underlying factor [5] and population aging, a universal phenomenon that is more pronounced in higher-income countries, implies that CHF prevalence will continue to increase [14,15,16]. In this context of advanced age, multimorbidity, and frailty, the spectrum of cardiac and non-cardiac diseases associated with CHF is very broad and requires a comprehensive clinical approach [3]. This changing situation requires new diagnostic and therapeutic strategies that are realistic and adapted to the local setting [14]. However, these changing circumstances are not always taken into account. For example, clinical trials do not often adequately include this type of patient, which may limit the appropriate use of new drugs [17]. What’s more, the left ventricular ejection fraction (LVEF), the parameter most commonly used to evaluate patients with CHF, does not correlate well with multimorbidity, which is high in all cases but with a higher prevalence of noncardiac comorbidities in patients with preserved LVEF [18]. Furthermore, LVEF does not demonstrate a consistent association with mortality [19]. Therefore, new approaches in addition to the classic approaches are needed. This study shows that although the number of underlying diseases is not very high, their relative frequency has changed, the combinations between them have become increasingly complex, and the resulting clinical pictures differ according to age and sex. The reasons for these changes are beyond this study’s scope, but the data suggest they may be related to an increase in the age of CHF onset, improvement in previous care, and perhaps local determinants. However, the consequences are very significant, as doctors must treat different and more complex patients, which changes their therapeutic management, the resources required to treat them, and morbidity and mortality.

This work found an increase of up to six years in the age of first admission for CHF over the study period. In that same period, life expectancy at birth in Galicia, Spain, increased by 3.12 years to 80.44 years for men and 86.45 years for women in 2022 [20]. Likewise, there has been a gradual increase in the proportion of women; indeed, women have been the majority of new cases for the last 20 years. Taken together, these facts indicate a gradual shift in the patient profile toward older women [21]. Different studies have reported notable differences in CHF management between men and women [6,21,22,23,24]. For example, women are more likely to receive treatment that worsens CHF and are less likely to have cardiac studies performed [6]. This could be related to specific barriers in some countries [6] or to the most recent diagnostic criteria and the most innovative treatments in patients with heart failure with preserved ejection fraction [25]. This study’s data indicate that differences may be in part due to the older age and greater multimorbidity in women and the clinical and demographic differences associated with gender [3].

This study shows that the number of underlying chronic diseases in incident cases has almost doubled in just 20 years. Although it is known that the burden of multimorbidity is high in all cases [8], it differs according to sex [3,25,26]. This suggests that the different chronic diseases are not associated by chance, but rather are part of age- and sex-linked clusters [27]. This situation, which is independent of the patient’s ejection fraction, means that the prognosis during hospitalization will differ according to gender [28,29].

Another noteworthy finding is the significant increase in the number of acute diseases diagnosed during hospital admission (Table 1). This fact, which to the authors’ knowledge has not been previously reported, includes diseases that can be precipitating factors of CHF (e.g., infection of any origin) and those derived from congestion (e.g., hyponatremia). It could be claimed that this increase was partially secondary to differences in coding at different times during the follow-up period. However, this seems unlikely, as changes in the coding team were minimal during this period. In the authors’ opinion, this is more likely related to older individuals’ greater likelihood of having more diseases or having new ones triggered after an initial major health problem. Consequently, it is a new factor that increases patient complexity and difficulties in their management in clinical practice. This entails significant changes in the management and follow-up on these patients.

Taken together, these data suggest that as age and the proportion of women increase, the number of concomitant diseases increases, with a change, at least partially, in the most common diseases found in previous periods. The increased presence of some diseases does not mean that more traditional ones have disappeared, but rather that their relative frequency has changed (Table 3). For example, there is an increased prevalence of atrial fibrillation and depression in admissions for CHF in more recent periods [30]. Although both diseases are associated with more advanced stages of CHF, their greater frequency could also be related to changes in age and gender. Another issue is discerning which diseases were known prior to admission and what the temporal sequence of their onset was in the different time periods (ordering them according to greatest support). Other studies have observed significant changes in the prevalence of different diseases associated with CHF, such as ischemic heart disease [31]. Indeed, similar trends have been observed in studies conducted in different countries, although with marked regional disparities [8,14,16,31,32,33,34].

This study has several strengths and limitations. The strengths include the large number of patients included, the exhaustive data collection, and the long time period analyzed. On the other hand, there are certain limitations. First, it could be claimed that the data collection was not as exhaustive in the study’s first years, and thus the differences were merely the result of incomplete data collection. However, an administrative database of a single center was used, and the same criteria were always followed. What’s more, it has already been evaluated in previous studies. Therefore, this possible limitation is mitigated, though the study’s generalizability may be more limited. Second, the retrospective design could raise doubts about the quality and veracity of clinical information included for analysis, since the data source was an administrative database. However, the study was conducted based on the medical records made by attending physicians, and the subsequent coding was independently verified by researchers who are themselves experienced physicians. Third, not having the LVEF may be an important shortcoming that could limit this study’s interpretation. However, the study started when this parameter did not have the weight it has today in the classification and clinical management of these patients. Furthermore, multimorbidity is high and heterogeneous in all types of ventricular ejection fraction. Finally, the data are from a single hospital in Spain. The results cannot necessarily be extrapolated to other geographic regions or hospitals of different sizes; conducting similar studies in other health districts in Spain would thus be of interest.

In summary, this single-center study shows that the presentation of new cases of CHF has changed over time. Cases are more complex, there is a predominance of women, more advanced age, and more pronounced multimorbidity with an increase in noncardiovascular diseases and concomitant acute diseases. This will entail very significant changes in the management, routine clinical practice, and follow-up of these patients. More studies are needed on the local level as these changes may differ according to geographic areas.

## Figures and Tables

**Table 1:** Clinical characteristics according to periods.

	Period 2000–2004	Period 2005–2009	Period 2010–2014	Period 2015–2019	Period 2020–2022	*p*
No. of cases	7059	4697	3674	3238	1059	
Age (years)	75.9 ± 11.2	77.4 ± 10.9	78.9 ± 11.4	81.4 ± 11.7	81.6 ± 11.5	0.0001
Gender (male)	51.7	49.5	49.1	45.7	48.6	0.0001
Acute diseases	1.1 ± 1.4	1.4 ± 1.6	3.5 ± 3.8	3 ± 3	2.7 ± 2.5	0.0001
Chronic diseases	3.6 ± 1.9	4.1 ± 2.1	7.7 ± 3.5	6.4 ± 3.5	6.5 ± 2.6	0.0001
Readmissions	4.8 ± 4.2	4 ± 3	3.2 ± 2.4	2.4 ± 1.7	1.5 ± 0.9	0.0001
Follow-up time (days)	2845.2 (2248.1)	2559.7 (1773.4)	1707.8 (1221.2)	881.1 (718.9)	291.9 (255.8)	0.0001

Continuous variables expressed as mean ± SD; Categorical variables expressed as %.

**Table 2:** Diagnoses and Items by Period.

	Period 2000–2004	Period 2005–2009	Period 2010–2014	Period 2015–2019	Period 2020–2022
No. of cases	7059	4697	3674	3238	1059
No. of transactions	30,344	17,829	11,198	7540	1539
No. of diagnoses	252	251	245	238	207
Diagnoses per patient (median)	3	4	5	5	7
Itemset	218	354	1134	1315	4241
Majority No. items/itemset (%)	2 Items (51.4%)	2 Items (52.8%)	3 Items (37.1%)	3 Items (40.3%)	4 Items (34.2%)
Items (median)	2	2	3	3	3
Items (mean)	1.75	1.95	2.77	2.82	3.44

**Table 3:** Rules of association.

Rules of Association 2000–2004			Support	Confidence	Lift	Number
{Cardiomyopathy}	=>	{Congestive heart failure}	0.0191	0.7208	1.5891	581
{Pulmonary hypertension}	=>	{Congestive heart failure}	0.025	0.5958	1.3133	759
{Valvular heart disease, Arrhythmias}	=>	{Congestive heart failure}	0.0121	0.5919	1.3049	367
{Arrhythmias, Miscellaneous}	=>	{Congestive heart failure}	0.0268	0.5824	1.2838	813
{Valvular heart disease}	=>	{Congestive heart failure}	0.0431	0.5761	1.2701	1309
{Diabetes mellitus 2, Arrhythmias}	=>	{Congestive heart failure}	0.0107	0.5755	1.2686	324
{Other infections, Arrhythmias}	=>	{Congestive heart failure}	0.0128	0.5742	1.2657	387
{Acute kidney failure}	=>	{Congestive heart failure}	0.0393	0.5741	1.2656	1193
{Valvular heart disease, Miscellaneous}	=>	{Congestive heart failure}	0.0112	0.5736	1.2645	339
{Arrhythmias, chronic obstructive pulmonary disease}	=>	{Congestive heart failure}	0.0113	0.5736	1.2644	343
Rules of association 2005–2009			Support	Confidence	Lift	Number
{Chronic kidney disease, Acute respiratory failure}	=>	{Congestive heart failure}	0.0102	0.7583	1.5523	182
{Valvular heart disease, Pulmonary hypertension}	=>	{Congestive heart failure}	0.0121	0.7465	1.5281	215
{Arrhythmias, Acute kidney failure}	=>	{Congestive heart failure}	0.0126	0.7329	1.5002	225
{Pleural effusion}	=>	{Congestive heart failure}	0.0184	0.7321	1.4987	328
{Arrhythmias, Acute respiratory failure}	=>	{Congestive heart failure}	0.0121	0.7264	1.4868	215
{Acute respiratory failure, Acute renal failure}	=>	{Congestive heart failure}	0.0116	0.7263	1.4867	207
{Chronic kidney disease, Arrhythmias}	=>	{Congestive heart failure}	0.013	0.7108	1.4549	231
{Cardiomyopathy}	=>	{Congestive heart failure}	0.0175	0.7107	1.4548	312
{Anemia, Arrhythmias}	=>	{Congestive heart failure}	0.0121	0.7036	1.4402	216
{Pulmonary hypertension}	=>	{Congestive heart failure}	0.0335	0.6945	1.4217	598
Rules of association 2010–2014			Support	Confidence	Lift	Number
{Hypertension, Arrhythmias, Acute respiratory failure, Miscellaneous}	=>	{Congestive heart failure}	0.0112	0.9058	1.609	125
{Hypertension, Arrhythmias, Acute respiratory failure, Atrial fibrillation}	=>	{Congestive heart failure}	0.0102	0.8976	1.5945	114
{Hypertension, Acute respiratory failure, Atrial fibrillation}	=>	{Congestive heart failure}	0.0104	0.8931	1.5865	117
{Hypertension, Arrhythmias, Acute respiratory failure}	=>	{Congestive heart failure}	0.014	0.8771	1.558	157
{Arrhythmias, Acute respiratory failure, Miscellaneous}	=>	{Congestive heart failure}	0.0176	0.8717	1.5484	197
{Cardiomyopathies, Miscellaneous}	=>	{Congestive heart failure}	0.011	0.8662	1.5387	123
{Acute respiratory failure, Atrial fibrillation, Miscellaneous}	=>	{Congestive heart failure}	0.0138	0.8652	1.5368	154
{Arrhythmias, Acute respiratory failure, Atrial fibrillation, Miscellaneous}	=>	{Congestive heart failure}	0.013	0.8639	1.5346	146
{Arrythmias, Acute respiratory failure}	=>	{Congestive heart failure}	0.0278	0.8567	1.5219	311
{Valvular heart disease, Acute respiratory failure}	=>	{Congestive heart failure}	0.0127	0.8503	1.5104	142
Rules of association 2015–2019			Support	Confidence	Lift	Number
{Arrhythmias, Acute respiratory failure, Acute renal failure}	=>	{Congestive heart failure}	0.0123	0.949	1.44	93
{Chronic kidney disease, Arrhythmias, Acute respiratory failure}	=>	{Congestive heart failure}	0.0117	0.9263	1.4056	88
{Arrythmias, Acute respiratory failure}	=>	{Congestive heart failure}	0.0459	0.9081	1.378	346
{Arrhythmias, Acute respiratory failure, Miscellaneous}	=>	{Congestive heart failure}	0.0275	0.9039	1.3716	207
{Hypertension, Arrhythmias, Acute respiratory failure}	=>	{Congestive heart failure}	0.0218	0.9011	1.3673	164
{Valvular heart disease, Cardiomyopathies}	=>	{Congestive heart failure}	0.0102	0.8953	1.3586	77
{Other infections, Arrhythmias, Acute respiratory failure, Miscellaneous}	=>	{Congestive heart failure}	0.0164	0.8921	1.3537	124
{Other infections, Arrhythmias, Acute respiratory failure}	=>	{Congestive heart failure}	0.0252	0.892	1.3536	190
{Hypertension, Arrhythmias, Acute respiratory failure, Miscellaneous}	=>	{Congestive heart failure}	0.0147	0.888	1.3475	111
{Chronic kidney disease, Acute respiratory failure, Acute renal failure}	=>	{Congestive heart failure}	0.0156	0.8872	1.3463	118

The term ‘arrhythmias’ includes all arrhythmias except atrial fibrillation. Support: The relative frequency of comorbidities in the data set. **Confidence:** The conditional probability of the occurrence of itemset2 given itemset1. Lift: Measures the independence between itemset1 and itemset2. A value greater than 1 indicates that the antecedent itemset and the consequent itemset are dependent and positively correlated.

## Data Availability

Data available on request from the authors.

## Appendix A

**Table A1:** Rules of association.

Rules of Association 2000–2004			Support	Confidence	Lift	Number
{Cardiomyopathy}	=>	{Congestive heart failure}	0.0191	0.7208	1.5891	581
{Pulmonary hypertension}	=>	{Congestive heart failure}	0.025	0.5958	1.3133	759
{Valvular heart disease, Arrhythmias}	=>	{Congestive heart failure}	0.0121	0.5919	1.3049	367
{Arrhythmias, Miscellaneous}	=>	{Congestive heart failure}	0.0268	0.5824	1.2838	813
{Valvular heart disease}	=>	{Congestive heart failure}	0.0431	0.5761	1.2701	1309
{Diabetes Mellitus 2, Arrhythmias}	=>	{Congestive heart failure}	0.0107	0.5755	1.2686	324
{Other infections, Arrhythmias}	=>	{Congestive heart failure}	0.0128	0.5742	1.2657	387
{Acute kidney failure}	=>	{Congestive heart failure}	0.0393	0.5741	1.2656	1193
{Valvular heart disease, Miscellaneous}	=>	{Congestive heart failure}	0.0112	0.5736	1.2645	339
{Arrhythmias, chronic obstructive pulmonary disease}	=>	{Congestive heart failure}	0.0113	0.5736	1.2644	343
{Valvular heart disease, Hypertension}	=>	{Congestive heart failure}	0.0112	0.5721	1.2613	341
{Atrial fibrillation}	=>	{Congestive heart failure}	0.0143	0.5606	1.2357	435
{Chronic kidney disease}	=>	{Congestive heart failure}	0.0378	0.5584	1.231	1147
{Arrhythmias}	=>	{Congestive heart failure}	0.0799	0.5548	1.2231	2423
{Other pulmonary disease}	=>	{Congestive heart failure}	0.01	0.5497	1.2118	304
{Acute respiratory failure}	=>	{Congestive heart failure}	0.0432	0.5437	1.1986	1312
{Dementia}	=>	{Congestive heart failure}	0.0245	0.5319	1.1725	742
{Anemia}	=>	{Congestive heart failure}	0.0413	0.5208	1.148	1253
{Hypertension, Arrhythmias}	=>	{Congestive heart failure}	0.0239	0.5197	1.1456	726
{Abnormality on additional tests}	=>	{Congestive heart failure}	0.0194	0.5162	1.1379	590
{Diffuse Parenchymal Lung Disease}	=>	{Congestive heart failure}	0.0165	0.516	1.1374	501
{Drug adverse effects}	=>	{Congestive heart failure}	0.018	0.5156	1.1365	547
{Goiter}	=>	{Congestive heart failure}	0.0137	0.5142	1.1335	417
{Other kidney diseases}	=>	{Congestive heart failure}	0.012	0.507	1.1176	363
Rules of association 2005–2009			Support	Confidence	Lift	Number
{Chronic kidney disease, Acute respiratory failure}	=>	{Congestive heart failure}	0.0102	0.7583	1.5523	182
{Valvular heart disease, Pulmonary hypertension}	=>	{Congestive heart failure}	0.0121	0.7465	1.5281	215
{Arrythmias, Acute kidney failure}	=>	{Congestive heart failure}	0.0126	0.7329	1.5002	225
{Pleural effusion}	=>	{Congestive heart failure}	0.0184	0.7321	1.4987	328
{Arrhythmias, Acute respiratory failure}	=>	{Congestive heart failure}	0.0121	0.7264	1.4868	215
{Acute respiratory failure, Acute renal failure}	=>	{Congestive heart failure}	0.0116	0.7263	1.4867	207
{Chronic kidney disease, Arrhythmias}	=>	{Congestive heart failure}	0.013	0.7108	1.4549	231
{Cardiomyopathy}	=>	{Congestive heart failure}	0.0175	0.7107	1.4548	312
{Anemia, Arrhythmias}	=>	{Congestive heart failure}	0.0121	0.7036	1.4402	216
{Pulmonary hypertension}	=>	{Congestive heart failure}	0.0335	0.6945	1.4217	598
{Other mental disorders, Arrhythmias}	=>	{Congestive heart failure}	0.0104	0.6877	1.4078	185
{Arrhythmias, Hydronephrosis/Acute Urinary Retention}	=>	{Congestive heart failure}	0.0124	0.6863	1.4049	221
{Other infections, Arrhythmias}	=>	{Congestive heart failure}	0.0183	0.6579	1.3468	327
{Valvular heart disease, Arrhythmias}	=>	{Congestive heart failure}	0.0216	0.6559	1.3426	385
{Other infections, Chronic kidney disease}	=>	{Congestive heart failure}	0.011	0.6384	1.3069	196
{Valvular heart disease, Hypertension, Arrhythmias}	=>	{Congestive heart failure}	0.0103	0.6354	1.3007	183
{Chronic kidney disease, Acute renal failure}	=>	{Congestive heart failure}	0.0164	0.6342	1.2982	293
{Valvular heart disease}	=>	{Congestive heart failure}	0.0621	0.634	1.2978	1107
{Valvular heart disease, Miscellaneous}	=>	{Congestive heart failure}	0.0193	0.6335	1.2968	344
{Acute respiratory failure}	=>	{Congestive heart failure}	0.065	0.6233	1.2758	1158
{Myopericarditis}	=>	{Congestive heart failure}	0.0154	0.6222	1.2736	275
{Acute respiratory failure, miscellaneous}	=>	{Congestive heart failure}	0.018	0.6209	1.2709	321
{Arrhythmias, Chronic obstructive pulmonary disease}	=>	{Congestive heart failure}	0.0113	0.6204	1.2699	201
{Acute Renal failure, miscellaneous}	=>	{Congestive heart failure}	0.0127	0.6192	1.2674	226
{Hypertension, Acute respiratory failure}	=>	{Congestive heart failure}	0.011	0.6183	1.2656	196
{Arrhythmias, Miscellaneous}	=>	{Congestive heart failure}	0.04	0.6182	1.2654	714
{Diabetes Mellitus 2, Arrhythmias}	=>	{Congestive heart failure}	0.0153	0.6085	1.2456	272
{Other infections, Acute respiratory failure}	=>	{Congestive heart failure}	0.0173	0.6063	1.2411	308
{Valvular heart disease, Hypertension}	=>	{Congestive heart failure}	0.0214	0.6038	1.236	381
{Chronic kidney disease}	=>	{Congestive heart failure}	0.0545	0.6034	1.235	972
{Arrhythmias}	=>	{Congestive heart failure}	0.0992	0.6031	1.2346	1769
{Anemia, Chronic kidney disease}	=>	{Congestive heart failure}	0.0121	0.6022	1.2328	215
{Other infections, Acute renal failure}	=>	{Congestive heart failure}	0.0135	0.6015	1.2313	240
{Other endocrine disease, Atrial fibrillation}	=>	{Congestive heart failure}	0.0107	0.5969	1.2218	191
{Chronic kidney disease, Miscellaneous}	=>	{Congestive heart failure}	0.0133	0.5906	1.2089	238
{Other pulmonary disease}	=>	{Congestive heart failure}	0.019	0.5885	1.2047	339
{Anemia, Miscellaneous}	=>	{Congestive heart failure}	0.0148	0.5884	1.2044	263
{Acute kidney failure}	=>	{Congestive heart failure}	0.056	0.5871	1.2017	998
{Atrial fibrillation}	=>	{Congestive heart failure}	0.0273	0.5825	1.1924	487
{Hypertension, Arrhythmias}	=>	{Congestive heart failure}	0.0356	0.5695	1.1658	635
{Hypertension, Arrhythmias, Miscellaneous}	=>	{Congestive heart failure}	0.0171	0.569	1.1648	305
{Ischemic heart disease, Arrhythmias}	=>	{Congestive heart failure}	0.0124	0.5638	1.154	221
{Hydronephrosis/Acute Urinary Retention, Miscellaneous}	=>	{Congestive heart failure}	0.0118	0.5627	1.1518	211
{Dementia}	=>	{Congestive heart failure}	0.034	0.5616	1.1496	606
{Chronic respiratory failure}	=>	{Congestive heart failure}	0.0129	0.561	1.1483	230
{Adverse drug event}	=>	{Congestive heart failure}	0.0293	0.5607	1.1477	522
{Cardiac prosthesis or device}	=>	{Congestive heart failure}	0.0201	0.5508	1.1274	358
{Anemia}	=>	{Congestive heart failure}	0.0523	0.5466	1.1189	932
{Other endocrine disease, Miscellaneous}	=>	{Congestive heart failure}	0.0105	0.5374	1.0999	187
{Goiter}	=>	{Congestive heart failure}	0.0136	0.5352	1.0956	243
{Other infections, anemia}	=>	{Congestive heart failure}	0.0102	0.5339	1.0929	181
{Acute-on-chronic respiratory failure}	=>	{Congestive heart failure}	0.0164	0.528	1.0809	292
{Other mental disorders, Miscellaneous}	=>	{Congestive heart failure}	0.0108	0.5246	1.0738	192
{Dyslipidemia, Arrhythmias}	=>	{Congestive heart failure}	0.0132	0.5244	1.0735	236
{Social problems}	=>	{Congestive heart failure}	0.0179	0.5212	1.067	319
{Oxygen therapy dependence}	=>	{Congestive heart failure}	0.0132	0.521	1.0664	236
{Anemia, Hypertension}	=>	{Congestive heart failure}	0.0113	0.5206	1.0657	202
{Electrolyte disorders}	=>	{Congestive heart failure}	0.0342	0.5201	1.0646	609
{Hydronephrosis/Acute Urinary Retention}	=>	{Congestive heart failure}	0.0453	0.5193	1.0629	808
{Personal history of neoplasm}	=>	{Congestive heart failure}	0.0154	0.5159	1.0561	275
{Ischemic heart disease, Miscellaneous}	=>	{Congestive heart failure}	0.014	0.5155	1.0551	250
{Other kidney diseases}	=>	{Congestive heart failure}	0.0131	0.5143	1.0529	233
{Other infections, Miscellaneous}	=>	{Congestive heart failure}	0.0186	0.514	1.0521	331
{Diffuse Parenchymal Lung Disease}	=>	{Congestive heart failure}	0.0169	0.5119	1.0478	302
{Other endocrine disease}	=>	{Congestive heart failure}	0.0373	0.5076	1.0391	665
{Other infections, Hydronephrosis/Acute Urinary Retention}	=>	{Congestive heart failure}	0.022	0.5064	1.0367	393
{Asthma}	=>	{Congestive heart failure}	0.0132	0.5054	1.0345	235
{Other mental disorders}	=>	{Congestive heart failure}	0.0374	0.5053	1.0343	667
{Abnormality on additional tests}	=>	{Congestive heart failure}	0.0252	0.5017	1.0269	449
{Atherosclerosis}	=>	{Congestive heart failure}	0.0115	0.5012	1.026	205
Rules of association 2010–2014			Support	Confidence	Lift	Number
{Hypertension, Arrhythmias, Acute respiratory failure, Miscellaneous}	=>	{Congestive heart failure}	0.0112	0.9058	1.609	125
{Hypertension, Arrhythmias, Acute respiratory failure, Atrial fibrillation}	=>	{Congestive heart failure}	0.0102	0.8976	1.5945	114
{Hypertension, Acute respiratory failure, Atrial fibrillation}	=>	{Congestive heart failure}	0.0104	0.8931	1.5865	117
{Hypertension, Arrhythmias, Acute respiratory failure}	=>	{Congestive heart failure}	0.014	0.8771	1.558	157
{Arrhythmias, Acute respiratory failure, Miscellaneous}	=>	{Congestive heart failure}	0.0176	0.8717	1.5484	197
{Cardiomyopathies, Miscellaneous}	=>	{Congestive heart failure}	0.011	0.8662	1.5387	123
{Acute respiratory failure, Atrial fibrillation, Miscellaneous}	=>	{Congestive heart failure}	0.0138	0.8652	1.5368	154
{Arrhythmias, Acute respiratory failure, Atrial fibrillation, Miscellaneous}	=>	{Congestive heart failure}	0.013	0.8639	1.5346	146
{Arrythmias, Acute respiratory failure}	=>	{Congestive heart failure}	0.0278	0.8567	1.5219	311
{Valvular heart disease, Acute respiratory failure}	=>	{Congestive heart failure}	0.0127	0.8503	1.5104	142
{Chronic kidney disease, Arrhythmias, Acute renal failure}	=>	{Congestive heart failure}	0.0101	0.8496	1.5092	113
{Arrhythmias, Acute respiratory failure, Atrial fibrillation}	=>	{Congestive heart failure}	0.0166	0.8493	1.5087	186
{Acute respiratory failure, Atrial fibrillation}	=>	{Congestive heart failure}	0.018	0.8487	1.5076	202
{Dyslipidemia, Acute respiratory failure, Miscellaneous}	=>	{Congestive heart failure}	0.01	0.8358	1.4847	112
{Chronic kidney disease, Acute respiratory failure}	=>	{Congestive heart failure}	0.0162	0.8341	1.4816	181
{Valvular heart disease, Acute renal failure}	=>	{Congestive heart failure}	0.0126	0.8294	1.4733	141
{Ischemic heart disease, Acute renal failure}	=>	{Congestive heart failure}	0.0101	0.8248	1.4652	113
{Other infections, Arrhythmias, Acute respiratory failure}	=>	{Congestive heart failure}	0.01	0.8235	1.4629	112
{Chronic kidney disease, Arrhythmias, Miscellaneous}	=>	{Congestive heart failure}	0.0142	0.8196	1.4559	159
{Valvular heart disease, pulmonary hypertension}	=>	{Congestive heart failure}	0.0164	0.8142	1.4462	184
{Other endocrine disease, Cardiomyopathy}	=>	{Congestive heart failure}	0.01	0.8116	1.4417	112
{Chronic kidney disease, Arrhythmias, Atrial fibrillation}	=>	{Congestive heart failure}	0.0115	0.8063	1.4322	129
{Acute respiratory failure, acute renal failure}	=>	{Congestive heart failure}	0.0202	0.8014	1.4236	226
{Anemia, Arrhythmias, Miscellaneous}	=>	{Congestive heart failure}	0.014	0.801	1.4229	157
{Acute respiratory failure, Hydronephrosis/Acute Urinary Retention}	=>	{Congestive heart failure}	0.0129	0.8	1.4211	144
{Arrhythmias, Other pulmonary disease, Miscellaneous}	=>	{Congestive heart failure}	0.0121	0.8	1.4211	136
Rules of association 2015–2019			Support	Confidence	Lift	Number
{Arrhythmias, Acute respiratory failure, acute renal failure}	=>	{Congestive heart failure}	0.0123	0.949	1.44	93
{Chronic kidney disease, Arrhythmias, Acute respiratory failure}	=>	{Congestive heart failure}	0.0117	0.9263	1.4056	88
{Arrhythmias, Acute respiratory failure}	=>	{Congestive heart failure}	0.0459	0.9081	1.378	346
{Arrhythmias, Acute respiratory failure, Miscellaneous}	=>	{Congestive heart failure}	0.0275	0.9039	1.3716	207
{Hypertension, Arrhythmias, Acute respiratory failure}	=>	{Congestive heart failure}	0.0218	0.9011	1.3673	164
{Valvular heart disease, Cardiomyopathies}	=>	{Congestive heart failure}	0.0102	0.8953	1.3586	77
{Other infections, Arrhythmias, Acute respiratory failure, Miscellaneous}	=>	{Congestive heart failure}	0.0164	0.8921	1.3537	124
{Other infections, Arrhythmias, Acute respiratory failure}	=>	{Congestive heart failure}	0.0252	0.892	1.3536	190
{Hypertension, Arrhythmias, Acute respiratory failure, Miscellaneous}	=>	{Congestive heart failure}	0.0147	0.888	1.3475	111
{Chronic kidney disease, Acute respiratory failure, Acute renal failure}	=>	{Congestive heart failure}	0.0156	0.8872	1.3463	118
{Valvular heart disease, Acute respiratory failure}	=>	{Congestive heart failure}	0.0198	0.8869	1.3458	149
{Valvular heart disease, pulmonary hypertension}	=>	{Congestive heart failure}	0.0176	0.8867	1.3454	133
{Other infections, Mental disorders, Arrhythmias}	=>	{Congestive heart failure}	0.0114	0.8866	1.3453	86
{Anemia, Acute respiratory failure, Miscellaneous}	=>	{Congestive heart failure}	0.0103	0.8864	1.345	78
{Ischemic heart disease, Acute respiratory failure}	=>	{Congestive heart failure}	0.0145	0.8862	1.3447	109
{Dyslipidemia, Cardiomyopathies}	=>	{Congestive heart failure}	0.0113	0.8854	1.3435	85
{Diabetes mellitus 2, Arrhythmias, Acute respiratory failure}	=>	{Congestive heart failure}	0.0106	0.8791	1.334	80
{Pulmonary hypertension, Arrhythmias}	=>	{Congestive heart failure}	0.0143	0.878	1.3324	108
{Dyslipidemia, Arrhythmias, Acute respiratory failure}	=>	{Congestive heart failure}	0.013	0.875	1.3277	98
{Other infections, Acute respiratory failure, Acute renal failure}	=>	{Congestive heart failure}	0.0182	0.8726	1.3241	137
{Chronic kidney disease, Acute respiratory failure}	=>	{Congestive heart failure}	0.0135	0.8718	1.3229	102
{Cardiomyopathies, Arrhythmias}	=>	{Congestive heart failure}	0.0131	0.8684	1.3177	99
{Chronic kidney disease, Acute respiratory failure}	=>	{Congestive heart failure}	0.0292	0.8661	1.3143	220
{Other infections, Hypertension, Arrhythmias, Acute respiratory failure}	=>	{Congestive heart failure}	0.0127	0.8649	1.3124	96
{Other infections, Dementia, Arrhythmias}	=>	{Congestive heart failure}	0.0133	0.8621	1.3081	100
{Acute respiratory failure, Acute renal failure, Miscellaneous}	=>	{Congestive heart failure}	0.0149	0.8615	1.3073	112
{Acute respiratory failure, acute renal failure}	=>	{Congestive heart failure}	0.0338	0.8615	1.3072	255
{Other infections, Arrhythmias, Acute renal failure, Miscellaneous}	=>	{Congestive heart failure}	0.0115	0.8614	1.3071	87
{Other infections, Chronic kidney disease, Arrhythmias}	=>	{Congestive heart failure}	0.0139	0.8607	1.306	105
{Cardiomyopathy}	=>	{Congestive heart failure}	0.0325	0.8596	1.3044	245
{Cardiomyopathies, Miscellaneous}	=>	{Congestive heart failure}	0.0146	0.8594	1.304	110
{Anemia, Acute respiratory failure}	=>	{Congestive heart failure}	0.0227	0.855	1.2974	171
{Mental disorders, Hypertension, Arrhythmias}	=>	{Congestive heart failure}	0.0133	0.8547	1.2969	100
{Other infections, Arrhythmias, Acute renal failure}	=>	{Congestive heart failure}	0.0179	0.8544	1.2965	135
{Mental disorders, Arrhythmias}	=>	{Congestive heart failure}	0.0247	0.8532	1.2947	186
{Mental disorders, Arrhythmias, Miscellaneous}	=>	{Congestive heart failure}	0.013	0.8522	1.2931	98
{Electrolyte disorders, Hypertension, Arrhythmias}	=>	{Congestive heart failure}	0.0113	0.85	1.2898	85
{Other infections, Obesity, Arrhythmias}	=>	{Congestive heart failure}	0.0102	0.8462	1.284	77
{Other infections, Acute respiratory failure, Hydronephrosis/Acute Urinary Retention}	=>	{Congestive heart failure}	0.0107	0.8438	1.2803	81
{Other infections, Ischemic heart disease, Miscellaneous}	=>	{Congestive heart failure}	0.0105	0.8404	1.2753	79
{Electrolyte disorders, Valvular heart disease}	=>	{Congestive heart failure}	0.0111	0.84	1.2746	84
{Pulmonary hypertension}	=>	{Congestive heart failure}	0.0381	0.8392	1.2734	287
{Other infections, Anemia, Arrhythmias}	=>	{Congestive heart failure}	0.0138	0.8387	1.2727	104
{Obesity, Valvular heart disease}	=>	{Congestive heart failure}	0.0143	0.8372	1.2704	108
{Other infections, Ischemic heart disease, Miscellaneous}	=>	{Congestive heart failure}	0.0163	0.8367	1.2697	123
{Hypothyroidism, Arrhythmias}	=>	{Congestive heart failure}	0.0115	0.8365	1.2694	87
{Obesity, Hypertension, Arrhythmias}	=>	{Congestive heart failure}	0.0183	0.8364	1.2691	138
{Other infections, Chronic kidney disease, Acute respiratory failure}	=>	{Congestive heart failure}	0.0142	0.8359	1.2685	107
{Obesity, Arrhythmias}	=>	{Congestive heart failure}	0.029	0.8327	1.2635	219
{Other infections. Vascular heart disease, Arrhythmias}	=>	{Congestive heart failure}	0.0145	0.8321	1.2626	109
{Obesity, Acute respiratory failure, Miscellaneous}	=>	{Congestive heart failure}	0.0105	0.8316	1.2618	79
{Pleural effusion}	=>	{Congestive heart failure}	0.0261	0.8312	1.2613	197
{Diabetes mellitus 2, Acute respiratory failure}	=>	{Congestive heart failure}	0.0245	0.8296	1.2588	185
{Other infections, Hypothyroidism}	=>	{Congestive heart failure}	0.0102	0.828	1.2563	77
{Other endocrine disease, Acute respiratory failure.}	=>	{Congestive heart failure}	0.0159	0.8276	1.2558	120
{Hypertension, Cardiomyopathies}	=>	{Congestive heart failure}	0.0114	0.8269	1.2548	86
{Acute respiratory failure, Hydronephrosis/Acute Urinary Retention}	=>	{Congestive heart failure}	0.0183	0.8263	1.2539	138
{Electrolyte disorders, Valvular heart disease}	=>	{Congestive heart failure}	0.0113	0.8252	1.2522	85
{Other infections, Mental disorders, Arrhythmias}	=>	{Congestive heart failure}	0.0113	0.8252	1.2522	85
{Valvular heart disease, Chronic kidney disease}	=>	{Congestive heart failure}	0.026	0.8235	1.2496	196
{Anemia, Hypertension, Arrhythmias}	=>	{Congestive heart failure}	0.0149	0.8235	1.2496	112
{Diabetes mellitus 2, Hypertension, Acute respiratory failure}	=>	{Congestive heart failure}	0.013	0.8235	1.2496	98
{Diabetes mellitus 2, Acute respiratory failure, Miscellaneous}	=>	{Congestive heart failure}	0.0129	0.822	1.2474	97
{Electrolyte disorders, Arrhythmias}	=>	{Congestive heart failure}	0.0237	0.8211	1.2459	179
{Mental disorders, Acute respiratory failure}	=>	{Congestive heart failure}	0.0188	0.8208	1.2455	142
{Hypertension, pulmonary hypertension}	=>	{Congestive heart failure}	0.0109	0.82	1.2443	82
{Electrolyte disorders, Acute respiratory failure}	=>	{Congestive heart failure}	0.0223	0.8195	1.2435	168
{Acute respiratory failure, social problems}	=>	{Congestive heart failure}	0.0156	0.8194	1.2434	118
{Other infections. Vascular heart disease, Arrythmia, Miscellaneous}	=>	{Congestive heart failure}	0.0102	0.8191	1.243	77
{Hypertension, Acute respiratory failure, Miscellaneous}	=>	{Congestive heart failure}	0.0228	0.819	1.2428	172
{Other infections, Arrhythmias}	=>	{Congestive heart failure}	0.0675	0.8183	1.2417	509
{Hypertension, Ischemic heart disease, Arrhythmias}	=>	{Congestive heart failure}	0.0155	0.8182	1.2415	117
{Other infections, Anemia, Acute respiratory failure}	=>	{Congestive heart failure}	0.0107	0.8182	1.2415	81
{Anemia, Valvular heart disease, Miscellaneous}	=>	{Congestive heart failure}	0.0101	0.8172	1.24	76
{Ischemic heart disease, Arrhythmias, Miscellaneous}	=>	{Congestive heart failure}	0.0178	0.8171	1.2398	134
{Acute respiratory failure, miscellaneous}	=>	{Congestive heart failure}	0.0525	0.8165	1.239	396
{Arrhythmias, Social problems, Miscellaneous}	=>	{Congestive heart failure}	0.0123	0.8158	1.2379	93
{Other infections, Hypertension, Arrhythmias}	=>	{Congestive heart failure}	0.0363	0.8155	1.2374	274
{Chronic kidney disease, Ischemic heart disease}	=>	{Congestive heart failure}	0.018	0.8144	1.2357	136
{Hypertension, Arrhythmias, Chronic obstructive pulmonary disease}	=>	{Congestive heart failure}	0.0122	0.8142	1.2354	92
{Dementia, Arrhythmias, Miscellaneous}	=>	{Congestive heart failure}	0.0139	0.814	1.2351	105
{Other infections, Arrhythmias, Miscellaneous}	=>	{Congestive heart failure}	0.0427	0.8131	1.2339	322
{Other infections, Electrolyte disorders, Acute respiratory failure}	=>	{Congestive heart failure}	0.0103	0.8125	1.2329	78
{Valvular heart disease, Hypertension, Arrhythmias}	=>	{Congestive heart failure}	0.0276	0.8125	1.2329	208
{Chronic kidney disease, Arrhythmias}	=>	{Congestive heart failure}	0.0424	0.8122	1.2324	320
{Arrhythmias, Social problems}	=>	{Congestive heart failure}	0.0195	0.8122	1.2324	147
{Diabetes mellitus 2, Dyslipidemia, Acute respiratory failure}	=>	{Congestive heart failure}	0.0109	0.8119	1.232	82
{Chronic kidney disease, Arrhythmias, Miscellaneous}	=>	{Congestive heart failure}	0.0239	0.8108	1.2303	180
{Other infections, Arrhythmias, Chronic obstructive pulmonary disease}	=>	{Congestive heart failure}	0.0102	0.8105	1.2299	77
{Pulmonary hypertension, Miscellaneous}	=>	{Congestive heart failure}	0.0158	0.8095	1.2284	119
{Dementia, Arrhythmias}	=>	{Congestive heart failure}	0.0236	0.8091	1.2277	178
{Dementia, Acute respiratory failure}	=>	{Congestive heart failure}	0.0219	0.8088	1.2273	165
{Valvular heart disease, Arrhythmias}	=>	{Congestive heart failure}	0.0488	0.8088	1.2273	368
{Other infections. Hypertension, Arrhythmias, Miscellaneous}	=>	{Congestive heart failure}	0.0247	0.8087	1.2271	186
{Hypertension, Arrhythmias, Social problems}	=>	{Congestive heart failure}	0.0106	0.8081	1.2262	80
{Electrolyte disorders, Arrhythmias, Miscellaneous}	=>	{Congestive heart failure}	0.0139	0.8077	1.2256	105
{Valvular heard disease, Hypertension, Arrhythmias, Miscellaneous}	=>	{Congestive heart failure}	0.0172	0.8075	1.2252	130
{Other infections, Diabetes mellitus 2, Arrhythmias}	=>	{Congestive heart failure}	0.0145	0.8074	1.2252	109
{Obesity, Hypertension, Arrhythmias, Miscellaneous}	=>	{Congestive heart failure}	0.0117	0.8073	1.2251	88
	=>	{Congestive heart failure}	0.0277	0.8069	1.2245	209
{Dyslipidemia, Ischemic heart disease, Arrhythmias}	=>	{Congestive heart failure}	0.0127	0.8067	1.2241	96
{Valvular heard disease, Arrhythmias, Miscellaneous}	=>	{Congestive heart failure}	0.0292	0.8059	1.2228	220
{Obesity, Acute respiratory failure}	=>	{Congestive heart failure}	0.0192	0.8056	1.2224	145
{Valvular heart disease, Chronic kidney disease, Arrhythmias}	=>	{Congestive heart failure}	0.0121	0.8053	1.222	91
{Valvular heart disease, Ischemic heart disease}	=>	{Congestive heart failure}	0.017	0.805	1.2216	128
{Other infections, Arrhythmias, Hydronephrosis Acute Urinary Retention}	=>	{Congestive heart failure}	0.017	0.805	1.2216	128
{Chronic kidney disease, Ischemic heart disease, Miscellaneous}	=>	{Congestive heart failure}	0.0103	0.8041	1.2202	78
{Electrolyte disorders, Arrhythmias, Acute renal failure}	=>	{Congestive heart failure}	0.0103	0.8041	1.2202	78
{Obesity, Chronic kidney disease}	=>	{Congestive heart failure}	0.0135	0.8031	1.2187	102
{Mental disorders, Chronic kidney disease}	=>	{Congestive heart failure}	0.0141	0.803	1.2185	106
{Dyslipidemia, Acute respiratory failure, Miscellaneous}	=>	{Congestive heart failure}	0.0141	0.803	1.2185	106
{Obesity, Arrhythmias, Miscellaneous}	=>	{Congestive heart failure}	0.0172	0.8025	1.2177	130
{Arrhythmias, Abnormality on additional tests}	=>	{Congestive heart failure}	0.0188	0.8023	1.2174	142
{Arrythmias, Acute kidney failure}	=>	{Congestive heart failure}	0.0412	0.8015	1.2163	311
{Hypertension, Arrhythmias, Acute renal failure}	=>	{Congestive heart failure}	0.0139	0.8015	1.2162	
{Hypertension, Acute respiratory failure}	=>	{Congestive heart failure}	0.0452	0.8005	1.2146	341
{Valvular heart disease, Chronic obstructive pulmonary disease}	=>	{Congestive heart failure}	0.0106	0.8	1.2139	80
{Chronic kidney disease, Chronic obstructive pulmonary disease}	=>	{Congestive heart failure}	0.0122	0.8	1.2139	92
{Diabetes mellitus 2, Obesity, Arrhythmias}	=>	{Congestive heart failure}	0.0101	0.8	1.2139	76
{Dyslipidemia, Anemia, Chronic kidney disease}	=>	{Congestive heart failure}	0.0101	0.8	1.2139	76

The term ‘arrhythmias’ includes all arrhythmias except atrial fibrillation. **Support:** The relative frequency of comorbidities in the data set. **Confidence:** The conditional probability of the occurrence of itemset2 given itemset1. Lift: Measures the independence between itemset1 and itemset2. A value greater than 1 indicates that the antecedent itemset and the consequent itemset are dependent and positively correlated.
